# Tendentious effects of automated and manual metagenomic DNA purification protocols on broiler gut microbiome taxonomic profiling

**DOI:** 10.1038/s41598-020-60304-y

**Published:** 2020-02-25

**Authors:** Gabor Fidler, Emese Tolnai, Aniko Stagel, Judit Remenyik, Laszlo Stundl, Ferenc Gal, Sandor Biro, Melinda Paholcsek

**Affiliations:** 1 Department of Human Genetics, Faculty of Medicine, University of Debrecen, Debrecen, Hungary; 2 Institute of Food Technology, Faculty of Agricultural and Food Sciences and Environmental Management, University of Debrecen, Debrecen, Hungary

**Keywords:** Biodiversity, Environmental impact

## Abstract

Here, we developed protocols to improve sensitivity, rigor and comparability of 16S rRNA gene amplification-based next-generation sequencing (NGS) results. A thorough study was performed by evaluating extraction efficiency with respect to the yield, purity, fragmentation of the purified DNA, and sequencing metrics considering the number of quality reads, amplicon sequence variants (ASVs), community structure and biodiversity. We identified batch-effects that significantly bias broiler gastrointestinal tract (GIT) community compositions and made recommendations to improve sensitivity, consistency, and cross-study comparability. We found that the purity of the extracted nucleic acid had a strong effect on the success rate of downstream library preparations. The preparation of stool bacterial suspensions from feces showed a significant positive influence on community biodiversity by enriching Gram-negative bacteria and cataloguing low abundant taxa with greater success than direct processing of fecal material. Applications relying on the automated Roche MagNa Pure 24 magnetic-bead based method provided results with high consistency therefore it seems to be the optimal choice in large-scale studies for investigating broiler GIT microbiota.

## Introduction

The rapidly growing world population has also intensified poultry production, which is predicted to produce about 130 million tons of chicken meat by 2020 according to the OECD/FAO reports^1^. Currently, avicultures are the most effective breeding systems of global protein production^2^. As the gastrointestinal tract (GIT) microbiota is a major determinant of intensive poultry growth, health and immune status through effects on nutrient digestion and absorption, exploring the bacterial phylogeny of the chicken GIT is of the utmost interest^3^.

NGS based metagenomic applications are able to overcome the limitations of traditional culture-based methods by classifying a multitude of formerly uncultivable microbiota and determining compositional profiles and dynamics of the microbial communities^4,5^.

Inappropriate DNA purification protocols can lead to errors in the estimation of microbiota community composition^6^. Furthermore, there is no standard approach or single best protocol for 16S rRNA marker gene-based metagenomics surveys. Different metagenomic applications may lead to overrepresentations of some taxa and the omition of those with low abundance. Nevertheless, the exact composition of the biospecimen may also influence the performance of applied protocols^7^. Therefore, the choice of proper metagenomic DNA extraction method is critical and has to be appropriate for the biological questions being asked^6–11^. Numerous studies indicate that the combination of multiple extraction procedures applied to a single microbial environment helps reduce taxa representation biases associated with individual methods^6,9,12^.

Currently, a number of metagenomic studies rely on a wide range of commercially available kits^13,14^. This is due to their short hands-on time and adaptability to robotic platforms when conducting high-throughput studies. Traditional, non-kit-based DNA purification approaches are also being used for cost-efficient purification of microbial DNA samples. In applications, sample homogenization, bacterial lysis and DNA purification techniques are important sources of technical variations, which can significantly distort the apparent composition, structure and diversity of the microbiota^11,13–16^.

Because of the fact that stool samples differ substantially, specimen homogenization is vital. Nevertheless, the choice of homogenization strategy can obscure biologically meaningful differences. Bacteria present in the fecal matrix are not free-floating single organisms, but prefer to live in surface-associated communities where they adhere to biomaterials on the basis of nutritional and protective benefits^17–20^. The disruption of the bacteria with rigid cell walls and different membrane structures affects the effectiveness of polymerase chain reaction (PCR)-mediated metagenomic approaches, therefore the choice of the method affects the quantity, quality, distribution and diversity of nucleic acid molecules^21–25^.

The principle of DNA separation methods (magnetic bead, silica column, precipitation) adapted to manual or automated platforms largely influences the yield of the target DNA and the removal of residual PCR inhibitors, which are sources of pitfalls in nucleic acid amplifications^21,22^. Furthermore, high degree of standardization can be achieved by using automated extraction platforms, which minimize inter-operator variability and hands-on time^19^. It has also been demonstrated that the yield and-, purity of extracted DNA are important determinants of the number of sequenced reads^23,24^.

It is challenging to achieve sufficient reproducibility in 16S rRNA gene based next-generation sequencing (NGS) surveys. Different metagenomic DNA purification methods have different biases, which can significantly impact the results. Therefore, it is important to compare different DNA purification protocols and establish their advantages and limitations. The current study was prompted by the lack of adequate methodologies of metagenomic DNA extractions from feces with biotic and abiotic contaminants.

## Results

### Study overview

**Figure 1:**
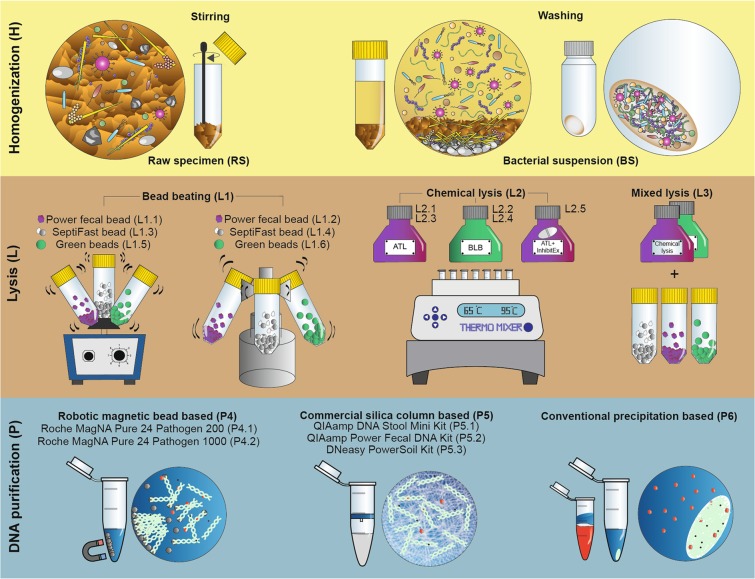
Detailed delineation of the different stages of metagenome DNA extraction approaches involving sample homogenization (H), lysis protocols (L), DNA purification methods (P). Direct lysis of the biologically diverse faces (raw specimen: RS) has the power to get access to both planktonic and sessile cells. Processing bacterial suspension: BS obtained by multiple washing steps free-floating bacteria can be separated from the indigestible compounds of the feces and manure particles. To compare the efficiency of lysis methods in disintegrating Gram-positive and Gram-negative cell boundaries, bead mill (L1), chemical cell disruption (L2) based techniques and the mixture of those (L3) were performed. To separate nucleic acids from sample lysates robotic magnetic bead (P4), manual silica column (P5) based commercial and manual nucleic acid precipitation based conventional (P6) techniques were used.

An overview of the technical variations associated with the three important stages of metagenomic nucleic acid extraction workflows such as sample homogenization (H), lysis protocol (L) and DNA purification (P) techniques is shown in Fig. 1. A detailed assemblage of the different metagenomic isolation methods including important technical variables is shown in Supplementary Fig. S1. Supplementary Table S1 indicates the standard methods (S1-S16) of metagenome DNA purifications that were used in the present study.

### General description of sequencing results

16S rRNA gene based (V3-V4 region) amplicon sequencing was carried out on Illumina MiSeq platform generating a total of 13.257.362 reads with the mean read count of 138.097 ± 31.408 (mean ± SD) reads per sample in a range of 77.997–382.901 reads. Quality filtering with the dada2 software resulted an average denoised read count of 70.987 ± 17.676 per sample and after the merging process the read count dropped to an average of 63395 ± 16586 reads per sample. At the end, the average of non-chimeric reads was 45748 ± 11107 per sample.

### DNA yield, quality, sequencing metrics and biodiversity

**Figure 2:**
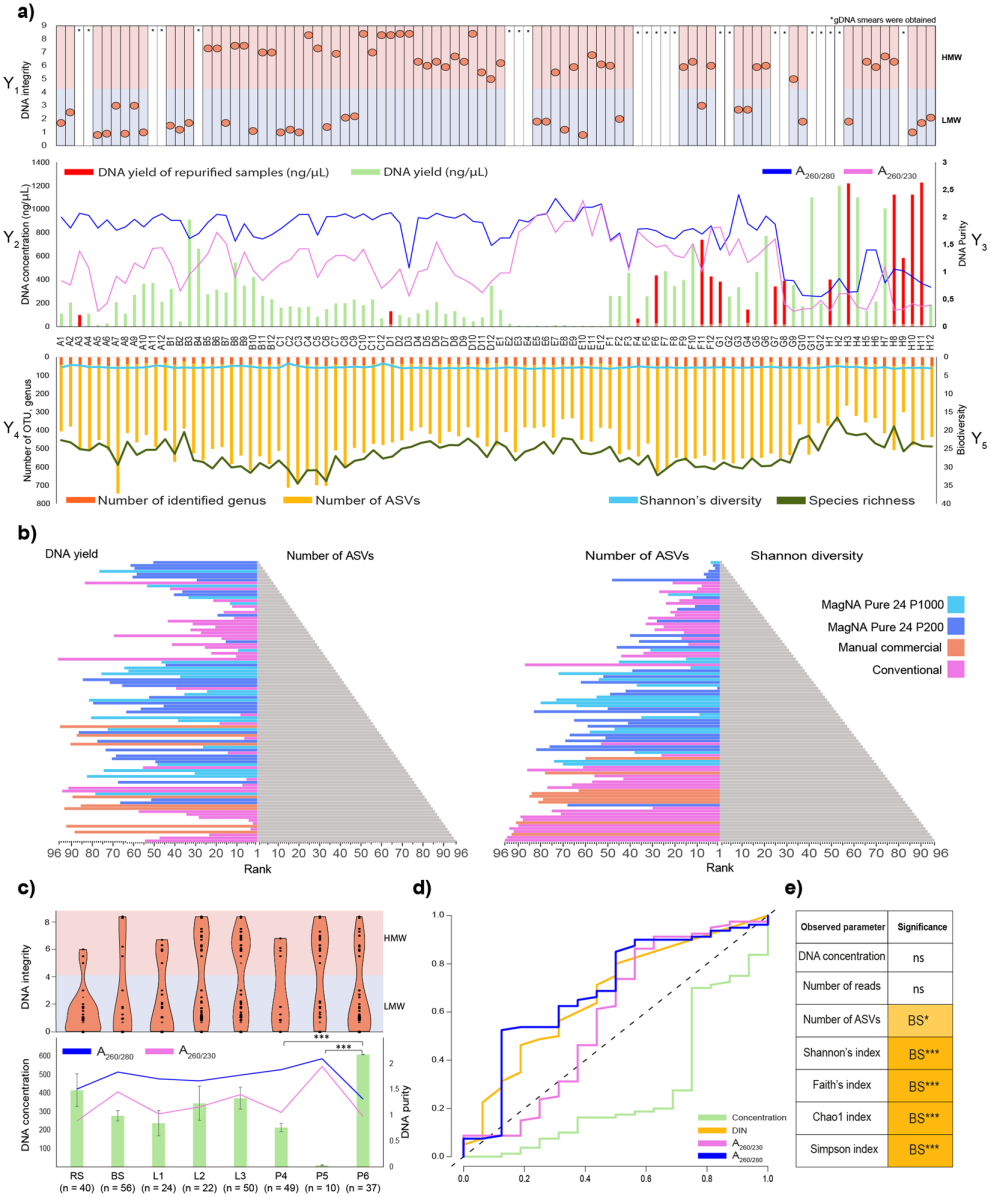
The effect of DNA yield and quality on downstream sequencing and observed community diversity. (**a**) On the Y1 axis of the DNA integrity (DIN) data are displayed for every sample elutes (A1-H12) sequestering high molecular weight (HMW) and low molecular weight (LMW) samples. Display of genomic DNA integrity (DIN) was done automatically by the TapeStation Analysis Software on the basis of the electropherogram intensity of the samples. Y2 axis represents total DNA yields (ng/µl). Total DNA extraction yields presented in ng/µl (bar-charts) of DNA per sample. Y3 axis shows the respective absorbance ratios A_260_/A_280_ and A_260_/A_230_ (line charts). Y4 axis presents sequencing metrics (number of identified genus, number of ASV). Y5 axis displays post-sequencing ecological inferences (Shannon’s diversity and species richness). (**b**) Pyramid plots display the results of rankings of every single method considering DNA yield in relation with the number of ASVs and obtained Shannon’s diversity indices. (**c**) Reports of DNA concentration and quality metrics from the point of view of the technical influential variables. Data shown are mean values with standard deviations. Violin plots represent the distribution of HMW vs. LMW DNA bands. (**d**) ROC represents the influence of extraction efficiency and suitability of the isolated nucleic acids quantities in downstream library preparation. (**e**) Significant differences observed in parameters between sample homogenizations. Asterisks report statistical significance; *P ≤ 0.05, **P ≤ 0.01, ***P ≤ 0.001, while “ns” stands for non-significance.

Several DNA extraction protocols have been tested for fecal microbiota community profiling. In 16.6% of the purified samples PCR amplification was inhibited. Importantly, in 87.5% (14 of 16) of these samples the phenol-chlorophorm method was applied (Fig. 2a). No apparent correlation was observed between DNA concentrations and the number of amplicon sequence variants (ASVs), whereas data rankings showed an obvious proportionality between the ASV numbers and the Shannon’s diversity indexes (Fig. 2b, Table S2). Based on these results, MagNa Pure 24 - Pathogen 200 Protocol seems to be an ideal choice to extract community DNA from bacterial suspensions and achieve high resolution of community composition with rare taxa.

### Sample homogenization and DNA purification have significant influence on DNA yield

The effects of sample homogenization, bacterial cell lysis and DNA purification methods on DNA concentrations are shown in Fig. 2c. For all tested parameters, the average concentration, quality and integrity of purified DNA were established. The highest DNA concentrations were obtained by feces homogenization by stirring (RS). In the case of RS, an average of ~1.5-fold increase in DNA yield was observed in comparison to bacterial cell suspensions (BS). Mixed lysis proved to be more effective than bead-beating resulting in ~1.57-times higher amount of nucleic acids. Conventional DNA precipitation methods resulted in the highest DNA yield (P < 0.0001) compared to the magnetic bead-based (3-fold increase) and silica column based (56-fold increase) methods. However, poor DNA quality (A_260_/A_230_ 1.3 ± 0.55) was observed in the majority of samples. The MagNa Pure 24 robotic platform managed to extract a large amount of good quality DNA, as indicated by optimal A_260_/A_280_ 1.8 ± 0.21–1.89 ± 0.2 absorbance ratios. The Qiagen kits were also able to generate acceptable values in the range of 1.8–2.2. No correlation was found between the fragmentation status of the metagenomic DNA (i.e. high molecular weight - HMW (DIN ≥ 4.5) versus fragmented low molecular weight - LMW (DIN < 4.5)) and yield or purity.

### The quantity and integrity of extracted nucleic acids have no effect on library preparation

Receiver-operating characteristic (ROC) analysis was performed with the outcome of amplicon PCR (succeeded, not succeeded) as the binary classifier. Area under the curve (AUC) values with 95% confidence intervals (95% CIs) and standard deviation (±SD) were calculated (Fig. 2d). Interestingly, absorbance ratios and shearing were associated with 2.4-times higher AUCs (mean AUC 0.64 ± 0.0822) than DNA concentration. For DNA concentration, the AUC was = 0.2746 (95% CI 0.1213–0.4280; P value of <0.004 ± 2.88), and for A_260_/A_280_ and A_260_/A_230_ absorbance ratios the AUCs were = 0.6910 (95% CI 0.5388–0.8432; P value of <0.013 ± 2.46) and 0.5820 (95% CI 0.3966–0.7674; P value of < 0.386 ± 0.86), respectively. DNA integrity (DIN) AUC was 0.6719 (95% CI 0.5244–0.8194; P value of <0.024 ± 2.28). From our data we conclude, that DNA purity has the strongest predictive power on successful amplicon PCR. Intriguingly, the lowest correlation was observed between the DNA concentration and successful downstream PCR amplification.

### Bacterial cell suspensions had a prosperous effect on the community diversity

Sample homogenization through the preparation of bacterial suspensions significantly improved the assessment of community diversity (Fig. 2e). These results are in line with our previous observations that pure DNA samples are conducive in obtaining diverse communities.

### Standard protocols strongly affect sequencing metrics and observed biodiversity

**Figure 3:**
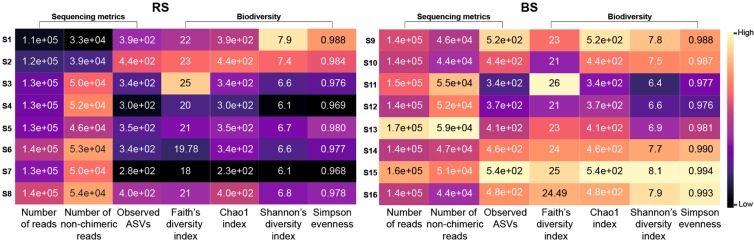
Overall performance of the concomitant batch-effects of 16 stool community DNA isolation approaches. S1-S16 designate metagenomic DNA isolation methodologies performed on raw specimens homogenized with stirring (RS) or washing to obtain bacterial cell suspensions (BS). Comparison of the sequencing and biodiversity metrics of the standard metagenome DNA purification methods. (S1, S9): MagNa Pure 24 Pathogen 200 protocol, (S2, S10): MagNa Pure 24 Pathogen 1000 protocol, (S3, S11): QIAamp DNA Stool Mini kit, (S4, S12): QIAmp Power Fecal kit, (S5, S13): Qiagen DNeasy Power Soil kit performing according to the manufacturer’s instructions. Performance of the conventional DNA isolation was also investigated coupled with different lysis protocols (S6, S14-bead-beating; S7, S15-chemical lysis; S8, S16-mixed lysis).

We evaluated the impact of 16 standard metagenome DNA isolation protocols [S1-S8 (RS), S9-S16 (BS)] on sequencing metrics (Supplementary Table S1). S1, S9, S2, S10 designate MagNa Pure 24 robotic methods, S3-S5, S11-S13 represent Qiagen kit-based manual methods, and S6-S8, S14-S16 denote conventional, phenol-chlorophorm DNA purification methods. Number of reads, non-chimeric reads, ASVs and community diversity metrics; Faith’s PD, Chao1 index, Shannon’s diversity indices, Simpson’s evenness are shown in Fig. 3. Conventional approaches coupled with BS consistently resulted in the highest community diversity especially when applying chemical (S15) or mixed (S16) lysis. Regarding the RS samples, S7 surprisingly performed the worst, whereas the highest biodiversity was achieved by S1, which at the same time generated the lowest read counts. This paradox was also observable in the case of S16 providing the second highest biodiversity and the lowest read counts. In the case of the BS samples, conventional DNA extraction approaches and the MagNa Pure 24 method with Pathogen 200 protocol resulted in high diversity estimates, while the commercial QIAmp kits S11, S12 recovered the least diverse communities. The Qiagen DNeasy Power Soil Kit produced similar diversities regardless of the sample homogenization method.

### Violating variations in overall taxonomic profiles

**Figure 4:**
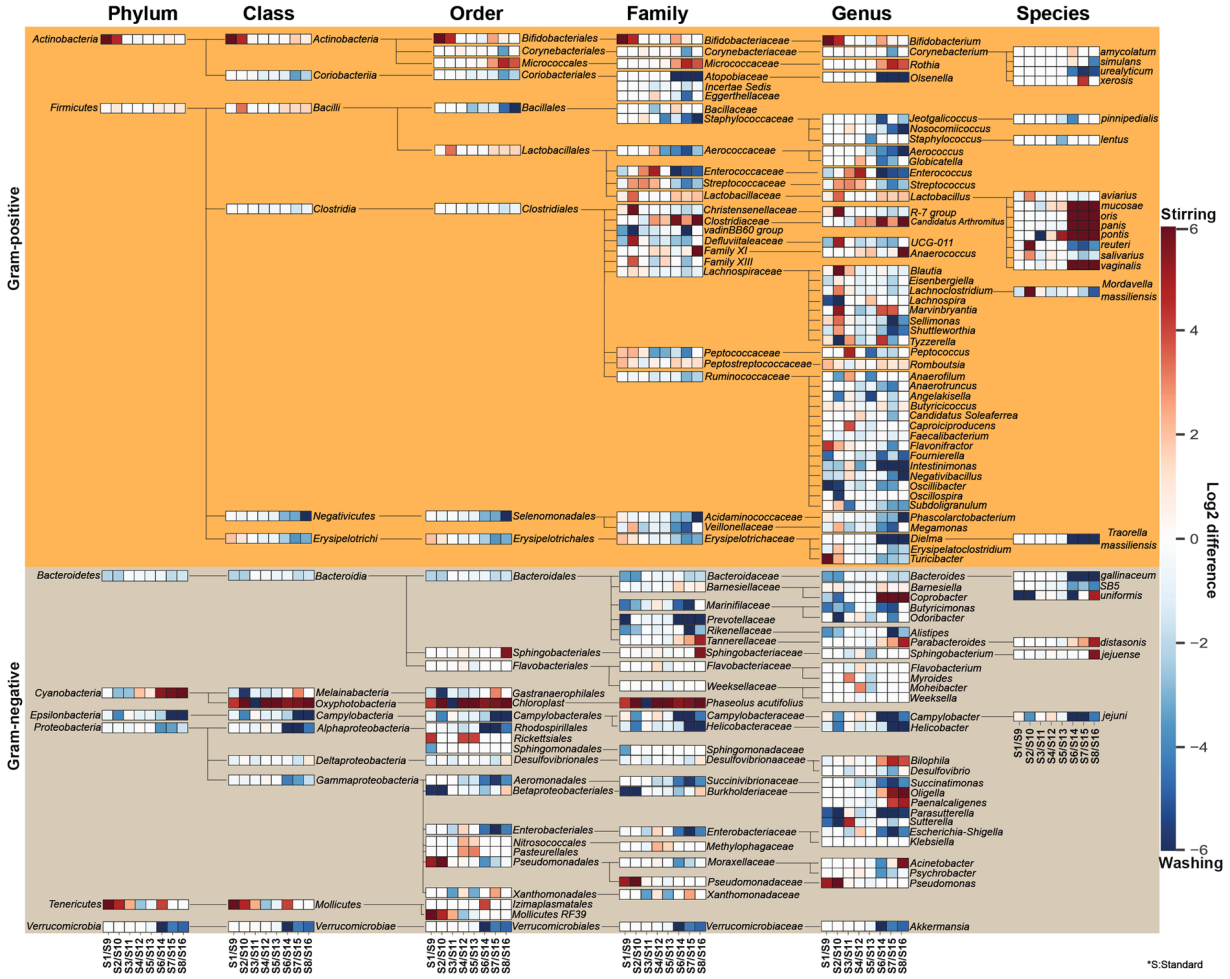
Remarkable abundance variations were observed in community structures. Composite heat map was created to pronounce the distortions in taxonomic profiles on the basis of normalized abundance data associated to eight standard metagenome DNA isolation methods for comparison of the impact of two different sample homogenization strategies (RS vs. BS). Extents of differences are illustrated on the composite heat-map with gradient colors where red scale represents dominance of taxa owed to processing raw specimens homogenized due to stirring; log2(RS/BS) >0 whereas blue scale represents the overrepresentation of taxa associated to bacterial suspensions; log2(RS/BS) <0. Significant shifts in abundances of the 143 taxa affected by the batch-effects can be observed.

The effects of sample homogenization (RS vs. BS) standard metagenome isolation approaches (S1-S8 vs. S9-S16) on observed community composition are shown in Fig. 4. Differences of normalized abundance data were attained by considering 8 phyla, 15 classes, 29 orders, 47 families, 72 genera and 22 species. Graphical representations were done by calculating the log2 differences in taxa abundances between RS - BS linked metagenome isolation approaches. Taxa with relative abundances lower than 1% were discarded from the analysis. Remarkably different taxonomic profiles were obtained when comparisons were made between subsets of RS vs. BS samples. This may be due in part to elevated concentrations of PCR inhibitors and nucleases in samples obtained from raw specimens compared to bacterial suspensions.

### Impact of batch-effects on beta-diversities

**Figure 5:**
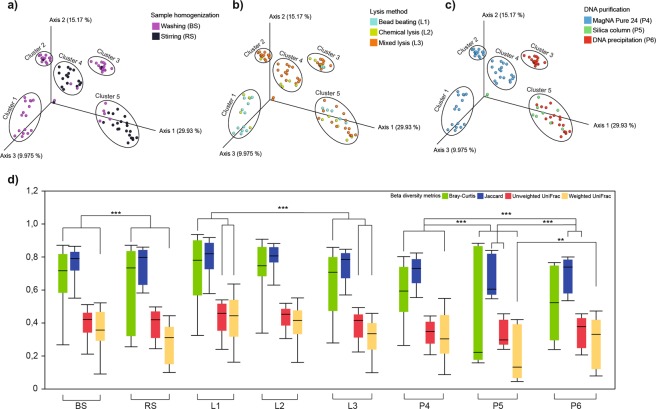
β-diversity distributions summarize the differences provoked by batch-effects related to metagenome DNA purification protocol variables. The summarization of the beta-diversity relationships is represented in three-dimensional scatter plots. Three principal coordinates plotted against each other summarizing the compositional differences among samples due to (**a**) sample homogenization (**b**) bacterial lysis (**c**) DNA purification. (**d**) Whisker plots represent the interquartile ranges (IQR) with min/maximum values and the median. Outlier values outside 1.5 times the interquartile range are omitted for clarity.

Principal coordinate analysis (PCoA) resulted in five cluster groups (cluster1 - cluster5) with different spatial ordinations. Different profiles were delineated in the case of BS (cluster1, 2, 3) in comparison to RS (cluster4, 5) samples (Fig. 5a). Dissimilarities in community compositions largely arise from the depletion of residents of sessile biofilm communities associated with solid organic particles in feces. In general, lysis protocols had no profound impact on the β-diversity profiles (Fig. 5b), except for MagNa Pure 24-based applications, where bead-beating based (cluster1) vs. mixed lysis techniques (cluster2, 4) showed a strong influence on differences in community compositions (Fig. 5b,c). Cluster3 displays a strong correlation between bacterial suspensions and DNA precipitation methods indicating a very high consistency. The choice of sample homogenization was not relevant in the case of silica membrane-based methods - P5 (Fig. 5a,c). Samples corresponding the robotic MagNa Pure 24 method were split into two subclasses with regards to sample homogenization (cluster1, 2 vs. cluster4), from which cluster2 exclusively represents bacterial suspensions where DNA was isolated with the Pathogen 200 protocol (Fig. 5c). Our observations based on PCoA plots were statistically strengthened by computing distance-based dissimilarity matrices to unravel protocol effects with significant influence on overall community variations (Fig. 5d). Bar lengths indicate within group compositional differences while between sample distances were calculated on the basis of quantitative (Bray-Curtis, weighted UniFrac) and qualitative (Jacquard, unweighted UniFrac) dissimilarity-based statistics.

### Detailed evaluation of the bacterial cell lysis protocols on DNA yield, purity and community diversity

**Figure 6:**
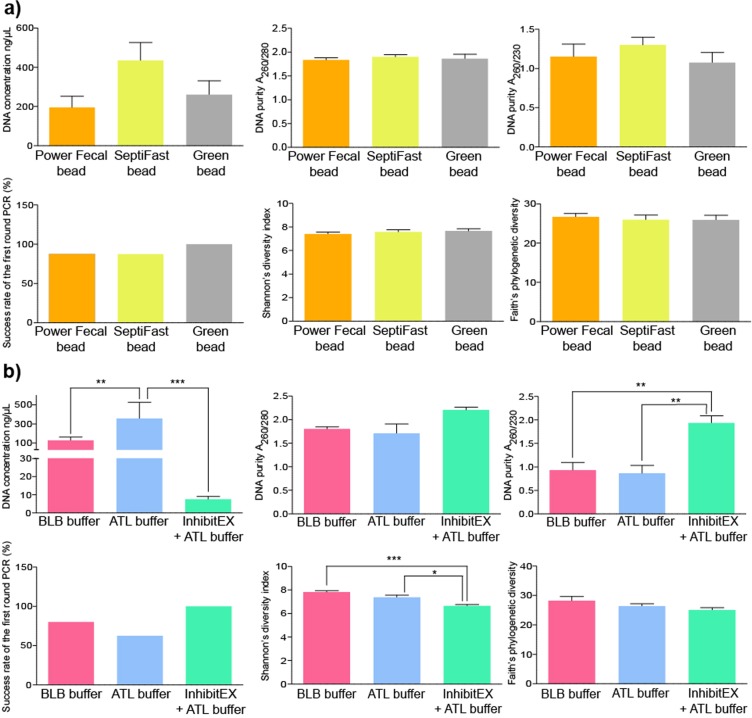
Comparison the effects of sample lysis. Bar charts displaying the effects of sample lysis on DNA yield (ng/ul), purity, downstream PCR amplification, estimated alpha diversity (Shannon’s index, ChaoI index, Faith’s PD) provoked by different (**a**) mechanical bead-mill based and (**b**) chemical lysis protocols.

We compared bead mill based and chemical lysis methods and estimated their effects on DNA yield, purity, downstream PCR amplification and biodiversity. Alterations in mechanical lysis techniques did not produce statistically significant differences (Fig. 6a). A remarkable gain in DNA amount was observed when samples were lysed by bead mills (326 ± 110 ng/µl) relative to chemical lysis (101 ± 148 ng/µl). To achieve chemical lysis, ATL, BLB buffers and the inhibitor absorbent InhibitEX Tablet were used (Fig. 6b). We found a significant positive association between the application of the ATL buffer and the DNA purification efficiency. Pretreatment with InhibitEX Tablet resulted in purified nucleic acids with reasonably good quality showing an overall positive effect on the purity and success rate of downstream PCR reactions, while exerting a detrimental effect on DNA yield. One can assume that nucleic acids prone to adsorb to the particles may be eliminated by the centrifugation step. Stool samples subjected to InhibitEX Tablet showed significantly lower Shannon’s diversity indexes, which may be associated with bacterial DNA loss. The use of both ATL and BLB buffers resulted in high quality and, yield of DNA. Strong positive correlation was observed between Shannon’s diversity and the application of the BLB and ATL.

### Significant shifts in Gram-profiles

**Figure 7:**
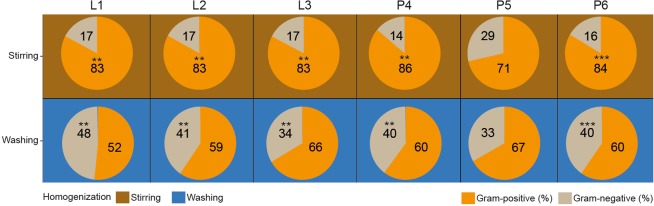
Profound sample homogenization mediated impact on Gram-distributions. (**a**) The aggregate effects of sample homogenizations joint to lysis protocols and DNA purification methods were estimated. (**b**) In the case of the different batch-effects Gram-positive and Gram-negative abundance comparisons were made between samples homogenized with stirring and washing. Asterisks report statistical significance; **P ≤ 0.01, ***P ≤ 0.001, while “ns” stands for non-significance.

Significant distortions were detected in the Gram-positive and Gram-negative distribution profiles due to sample homogenization strategies (Fig. 7a,b). Interestingly, homogenization of feces due to stirring (RS) represented constant Gram ratios (17% Gram-negative vs. 83% Gram-positive) irrespective of sample lysis. In the case of raw specimens, mild distortions were seen between the MagNa Pure 24 - P4 robotic and the conventional phenol-chlorophorm method - P6 (Gram-positive 86% vs. 83%, Gram-negative 14% vs. 16%), while profound alterations were introduced due to silica column methods - P5 (Gram-positive 71%, Gram-negative 29%). In the case of bacterial suspensions, diverse results were obtained. Bead mill-based mechanical lysis resulted in highly balanced Gram-positive (52%) vs. Gram-negative (48%) ratios. The choice of sample homogenization exerted the least profound effect on the Gram-distributions of stool bacteria when DNA was recovered with silica column-based methods (Gram-negative: 29% vs. 33% vs. Gram-positive: 71% vs. 67%).

### Distortions in the core microbiota

**Figure 8:**
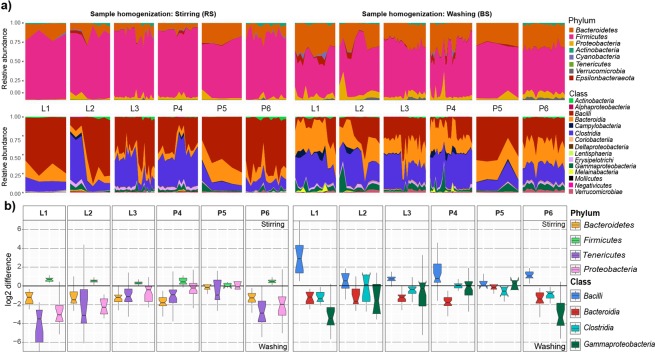
Complex representation/delineation of the dependence of the distribution of the core bacteria of broiler (*Gallus gallus domesticus*) according to different batch-effects of metagenome DNA extraction protocols. Distortions in the observed relative abundances on phylum and class level are shown. (**a**) Area plots show the relative abundance variations of the protocol variations in the 50% core microbiota compositions in relation to sample homogenization strategies. (**b**) Estimated log2 values of the proportions (stirring-RS/washing-BS) of abundance values associated with protocol batch-effects of dedicated phyla and classes showing clear distortions. The horizontal line is plotted at a value of 0 corresponding to equal proportions of values associated to differences in sample homogenizations. Box lengths indicate within sample variations.

To assess the effects of technical variables, core microbiomes were constructed by considering taxa represented in at least 50% of all sample aliquots. Complex comparisons in core bacteria distributions were made on all taxonomic ranks except species (Fig. 8). Different methods of sample homogenization resulted in pronounced differences in core microbiota abundances. Although the majority of the core microbiota fall into a limited number of phyla and classes, their relative proportions showed remarkable alterations (Fig. 8a). Four phyla (*Bacteroidetes, Firmicutes, Tenericutes, Proteobacteria*) and four classes (*Bacilli, Bacteroidia, Clostridia, Gammaproteobacteria*) displayed large differences in relative abundance. Fold changes of log2 abundance ratios (RS/BS) were used to estimate over- and under- representations of taxa (Fig. 8b). Homogenization by stirring resulted in increased Gram-positive members of the phyla *Firmicutes* (RS: 81.4% vs. BS: 60%), whereas bacterial suspensions provided higher abundance values for the Gram-negative *Bacteroidetes, Proteobacteria* and *Epsilonproteobacteria* (28.68% vs. 12.62%, 6.57%, vs. 3.06%, 1.9% vs. 0.69%) (Supplementary Table S3). On both taxonomic ranks the shortest box lengths were obtained with the Qiagen Kit based DNA separation methods (P5) indicating the highest concordance attained. Negligible alterations in abundance were observed for *Actinobacteria* (RS: 1.14% vs. BS: 1%), *Cyanobacteria* (RS: 0.54% vs. BS: 0.56%), *and Tenericutes* (RS: 0.2% vs. BS: 0.16%). In contrast, bacterial suspension favors *Verrucomicrobia* in conjunction with mixed lysis (BS: 1.27% vs. RS: 0.18%) or the phenol-chlorophorm method (BS: 2.62% vs. RS: 0.3%). More complex effects were observed on the class level. Homogenization by stirring resulted in increased representation of the major class *Bacilli* (RS: 56%) relative to bacterial suspensions (BS: 25.54%), but the latter favors prominent classes such as *Clostridia* (32.12% vs. 23.43%), *Bacteroidia* (28.68% vs. 12.62%) and *Gammaproteobacteria* (5.78% vs. 2.41%). Our results also demonstrate that commercial, silica column-based DNA isolation platforms introduce the least amount of variation in core community profiles.

### Technical artifacts and *in silico* taxonomic classification influence the proportion of quality reads and species resolution

**Figure 9:**
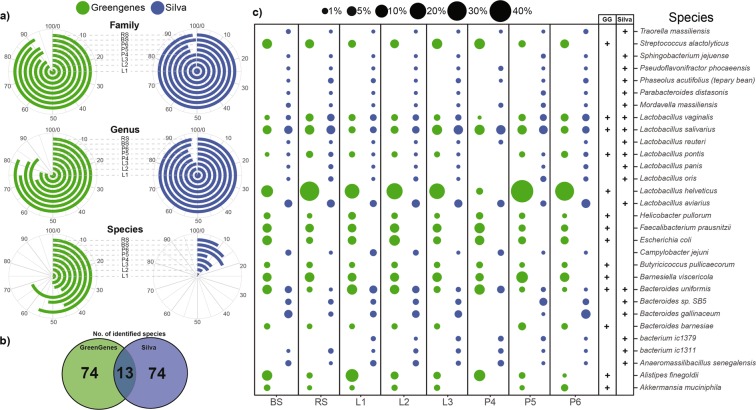
Dependence of the proportion of the classified reads across family and genus levels and remarkable ambiguity in the species resolution. (**a**) Polar plots show the percentages of the identified reads at family (L5), genus (L6) and species (L7) taxonomic levels bound to protocol manipulations. Angles of the radial coordinate systems represent the percentages of identified reads at dedicated taxonomic ranks. Increasing percentages are ordered in clockwise orientation. (**b**) Bubble charts are shortlisting the relative frequencies of the 30 most abundant species representing the species mining power of the Silva and GreenGenes annotation tools.

In addition to DNA extraction methods, technical aspects of *in silico* bioinformatic analyses also contribute to biases. We performed a comparison of metabarcoding analyses by using two sequencing databases; GreenGenes (GG) and Silva (S). At the phylum, class and order taxonomic levels, more than 99% of the reads have been successfully classified irrespective of the reference datasets. On average, Silva was able to rank more reads 97.95 ± 2.03% at the family level in comparison to GreenGenes (90.28 ± 2.84%) (Supplementary Table S4). The difference was more pronounced at the genus level (S: 95.99 ± 1.42 vs. GG: 78.86 ± 5.8%). As shown in Fig. 9a, the choice of sample processing (BS vs. RS) did not have a significant effect on the proportion of ranked reads (family: 98.91 ± 1.19%, genus: 97.05 ± 2.95) of the two taxonomic ranks. With Silva, the choice of metagenomic DNA purification method did not affect ranked at the family and genus levels significantly (94.67 ± 5.33%). Remarkable differences were observed between species ranking capacity of the two databases. On average, 87.12 ± 3.86% of the species were not traced by Silva, whereas GreenGenes was able to identify 51.74 ± 8.22% of the reads. The higher proportions of ranked reads at the species level showed no correlation with the number of the identified species (Fig. 9b). Silva and GG were able to identify a total of 87 species, but only 13 were identified by both databases. In 85.05% of the cases S and GG identified different species. Figure 9c represents the 30 most abundant species, of which only 4 species (*Lactobacillus vaginalis, Lactobacillus salivarius, Lactobacillus pontis, Bacteroides uniformis*) were detected by both S and GG. In these 4 cases, similar abundance values were calculated with the two databases. The use of GG led to significantly higher abundance estimates *for Streptococcus alactolyticus, Lactobacillus helveticus, Faecalibacterium prausnitzii, Escherichia coli, Barnesiella viscericola* and *Alistipes finegoldi*.

### Community taxonomy compositions suffer from biases of different kinds

**Figure 10:**
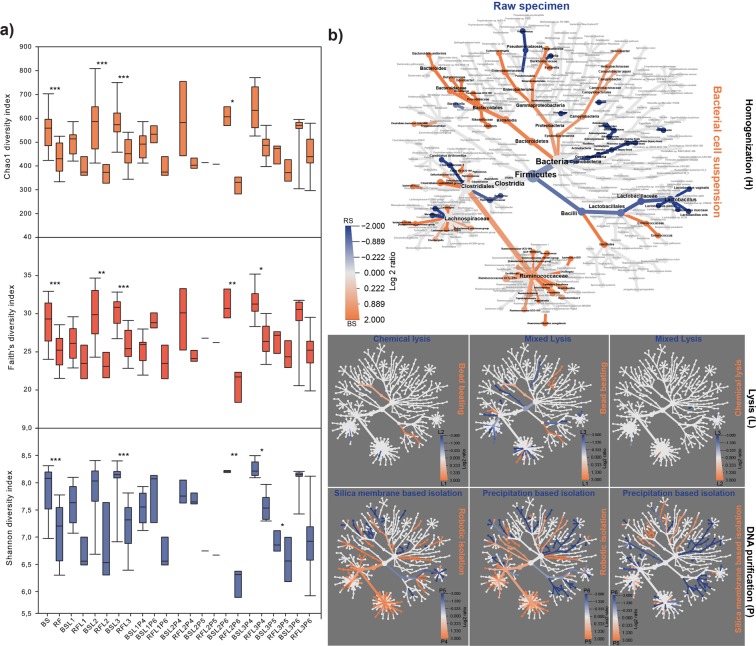
Quantitative and qualitative representation of the protocol batch-effects on community diversity and the taxonomic classifications. (**a**) Alpha-diversity distributions summarize the related to metagenome DNA purification protocol variables. Whisker plots represent the interquartile ranges (IQR) with min/maximum values with significance (*P ≤ 0.05, **P ≤ 0.01, ***P ≤ 0.001) and the median. (**b**) To unravel significant distortions a composite figure with seven community heat trees was made to represent the effects of correlated parameters of sample processing (H), lysis (L) and DNA purification (P) on community taxonomy. The annotated tree functions as a key for the unlabeled trees. Colored taxons represents the extents of log2 differences in taxa abundances. For instance, the annotated tree on the left allows a quantitative representation of diversity distortions by correlating the parameters of sample pre-processing (RS vs. BS) where taxa colored blue is enriched in RS samples shown in the column and those colored orange are enriched in BS shown in the row.

We quantified the cumulative effects of protocol variables sample processing (H; homogenization), lysis (L) and DNA purification (P) by assessing alpha-diversity distributions (Fig. 10a). Chemical lysis (L2) combined with MagNA 24 Nucleic Acid Purification (P4) and conventional precipitation-based techniques (P6) followed by bead-beating (L1) proved to be relatively robust to sample processing (BS vs. RS), but chemical lysis (L2) coupled with conventional precipitation-based methods (P6) was highly sensitive to different of homogenization.

To address the aggregate effects of technical variables on community taxonomy data heat trees were generated (Fig. 10b). Pairwise comparisons between parameters provide insights into the taxonomy shifts. To visualize differences between the massive data sets hierarchical structures of taxonomic classifications were developed, where the size of nodes and edges correlate with the abundance of clades. The taxonomic heat-trees reveal taxonomic lineages the detection of which may be challenging in the presence of a particular variable. Our analysis demonstrates, that the choice of sample preprocessing is of particular importance for the discrimination of lineages with relatively large nodes in orange (p_*Bacteroidetes*, p_*Proteobacteria*, c_*Bacteroidia*, c_*Gammaproteobacteria*, o_*Bacteroidales*, o_*Enterobacteriales*, f_*Ruminococcaceae*) and blue (p_*Firmicutes*, c_*Bacilli*, o_*Lactobacillales*, f_*Lactobacillaceae*, f_*Lachnospiraceae*). Grossly differences were observed between lineages from the aspect of the DNA preparation methods.

## Discussion

It is important to understand the potential sources of the intrinsic artifacts of 16S rRNA gene-based surveys especially for animal-associated microbiome methodologies which still lack concordance^26–30^. The effects of technical variables in protocols often exceed the biological effects of interest underlining the importance of minimizing these biases^31–37^.

In the present study we evaluated and compared parameters of metagenome DNA purification protocols by focusing on some of the methodological aspects known as protocol batch-effects including sample homogenization, lysis and DNA purification. Of note, our study did not evaluate the impact of alternative stool collection procedures with regards to starting volume, nucleic acid stabilizers, transportation, etc. and the effects of commonly used storage preservatives. By obtaining multiple sample aliquots of the same biological specimen (Fig. 1) we carried out parallel metagenomic DNA extractions and performed paired-end sequencing of the bacterial V3-V4 hypervariable regions of the 16S rRNA gene.

Inflated diversity can arise from ignoring the significance of reagent microbiome (“kitome”), which can greatly impact both 16S rRNA gene surveys and shotgun metagenomics due to the presence of contaminating bacteria in DNA extraction kits^38–41^. Its effect is ubiquitous and can vary between different lots of the same kit^41^. The empirical assessment of background contamination can be especially relevant and challenging in the case of low biomass samples. However, as DNA extraction kits introduce biases that affect all the samples equally, these biases can be controlled and mitigated by the use of the same batch of reagents and by including proper negative (“blank-swab”, “blank-extraction”, “blank-library”) and positive (“mock communities”) technical controls^40,41^.

We observed a considerable variation in the amount and fragmentation of community DNA resulting from protocol variables (Fig. 2). DNA shearing was found to be inconsequential to library preparation likely due to the use of relatively short amplicons. Importantly, we observed opposite effects of DNA quantity and quality on the success rate of downstream PCR amplification. On average, the conventional phenol-chlorophorm method yielded the highest gDNA concentrations and the worst purity resulting in a higher tendency for abortive amplicon PCR. This effect may be explained by elevated amounts of PCR inhibitors such as lipids, residual RNAs, polysaccharides, proteins, detergent contamination etc. To mitigate the effects of contaminants, additional bead-based DNA purification is strongly recommended. Our conclusion contrasts widely used metagenomic DNA library preparation protocols, which aim to maximize DNA concentration and maximize fragmentation^31^.

Based on the evaluation of eight standard metagenome DNA extraction methods we conclude that sample homogenization is the variable of greatest effect. We found that the method of choice has strong effects on A_260_/A_280_ absorbance ratios, number of quality reads, ASVs, observed biodiversity, and is the source of remarkable variability in community taxonomic profiles at every taxonomic level (Fig. 3). Among the DNA isolation methods tested, MagNa Pure 24 robotic isolation is the best to ensure the highest community diversity.

Standard methods (commercial silica membrane-based platforms) based on the use raw feces specimens (RS) and bead-mill lysis (L1) introduce bias in favor of the *Lactobacillales* and have a detrimental effect on estimates of community biodiversity. We believe that this is because biodiversity is proportionally influenced by the equilibrium of ASVs, with the abundant taxa generally increasing Simpson’s evenness thus decreasing Shannon entropy (Supplementary Fig. S2). Gram-positive *Bacilli* typically enrich biofilm forming clades, which show significantly higher abundance in RS samples likely due to physical rather than technical reasons. Sessile bacteria attached to manure particles have been depleted during multiple washing steps.

Our results clearly show that sample homogenization strategies such as stirring (RS) or washing (BS) have a profound effect on community taxonomy profiles. Remarkable differences in the abundance ratios were captured in the case of the Gram-positive *Lactobacillaceae* (RS: 52% vs. BS: 19%)*, Ruminococcaceae* (BS: 20% vs. RS: 12%)*, Enterobacteriaceae* (BS: 5% vs. RS: 1.4%) and Gram-negative *Bacteroidacea* (BS: 14% vs. RS: 6%), *Prevotellaceae* (BS: 2% vs. RS: <0.1%)*, Alcaligenaceae, Rickenellaceae, Pseudomonadaceae, Helicobacteriaceae, Desulfovibrionaaceae* and *Verrucomicrobiaceae* families when microbiota was isolated from raw stool specimens versus bacterial suspensions (Fig. 4). With regards to sequencing metrics, we detected an inverse relationship between read counts and ASV numbers.

Efficient lysis of bacterial cells with the removal of exo, and endonucleases and PCR inhibitors are critical for the proper purification of total nucleic acids from Gram-positive and Gram-negative stool-bacteria with heterogeneous cell wall structures. In search of the optimal DNA extraction method, we tested common bead-based and chemical lysis techniques and their combination (Fig. 6). With respect to bead mill-based lysis our results conflict with previous studies reporting significant loss in nucleic acid molecules bounded to the particles^42,43^. Our study demonstrated that bead-based lysis did not reduce, but in fact significantly enhanced DNA yield. Furthermore, this lysis method was not associated with a significant increase in alpha-diversity. Finally, our analyses suggest that the application of InhibitEX tablet beneficially influenced DNA purity, while significantly (P ≤ 0.001) decreasing DNA yield^9,20,22,31,44^. Using raw feces specimens, we observed no differences between lysis methods with respect to Gram distribution (G-positive: 83% vs. G-negative: 17%). However, in the case of bacterial suspensions mixed lysis was associated with the highest relative Gram-positive abundance (66%).

We also quantified and compared the effects of variables on distortions between communities. Stirring-based homogenization resulted in the over-representation of Gram-positive and under-representation of Gram-negative communities (P ≤ 0.001) (Fig. 7). These distortions are likely related to the differences between the growth modes of sessile (in particular *Enterococcus*, *Staphylococcus* of the Gram-positive phyla *Firmicutes*) and motile planktonic bacteria, and their pili and fimbriae colonizing biotic and abiotic surfaces of the poultry manure. We conclude that there is a strong positive correlation between the fraction of Gram-negatives and observed community diversity. This conclusion is consistent by our observation that bacterial suspensions are associated with significantly higher (P < 0.001) community diversity considering Shannon’s diversity, Faith’s, Chao and Simpson’s indexes, and allow better differentiation of Gram-negative taxa. However, our observations contrast with previous studies hypothesizing that lower relative abundance of Gram-positive bacteria would result in decreased diversity^31,45^. Nevertheless, handling with bacterial suspensions is a procedure which can be standardized, sequestering a remarkable amount of the biotic (plant debris) and abiotic contaminants of the fecal matter. In addition, cell counts can be easily estimated so making results more easily comparable being conducive in multi-center consortia studies.

From the point of view of extraction quality and protocol transferability the robotic MagNa Pure 24 Pathogen 200 and Pathogen 1000 isolation protocols proved to be superb, showing on average the highest biodiversity metrics (Faith’s PD 22.31 ± 2.12, Chao1: 439.98 ± 61.94, Shannon’s index: 7.50 ± 0.32). Furthermore, the phenol-chlorophorm DNA extraction and the MagNa Pure 24 200 protocol yielded the more reproducible results revealing highly diverse estimates on the stool microbiota. To our knowledge this was the first case implementing the robotic Roche MagNA Pure 24 DNA purification system (P4) for metagenomic purposes. Silica membrane-based DNA purification methods were associated with the lowest DNA concentrations, but good quality. They obtained high number of classified reads (family 95.04%, genus 94.79%) transmitting predominantly the most abundant taxa contributing to a general decline in community biodiversity (Fig. 9).

We compared the power of the of two *in silico* metabarcoding approaches (GreenGenes vs. Silva) to understand complex and taxonomically diverse samples. One obvious virtue of SILVA is that it is regularly curated while GreenGenes has not been updated since 2013. Remarkable inconsistency (85.05%) was shown between the results of the two sequencing annotation databases. This can be explained by the disadvantage that the V3-V4 variable regions of the 16S rRNA phylogenetic marker genes used as phylogenetic markers are very similar in sequence between non-closely related species^44,45^. This means that the lack of resolution of the different taxonomy datasets at species level is a biological issue which either classify the 16S rRNA genes correctly (true positive), do not classify them (false negative) or classify them wrongly (false positive)^46–48^.

With this methodological study we also catalogued the GIT microbiome of *Gallus gallus domesticus*, Ross 308 hybrid. Based on our observations, the phylum *Firmicutes* was overrepresented (70.34%), followed by *Bacteroides* (21.23%) and *Proteobacteria* (4.77%). These results are in accordance with results reported by Sofka *et al*. Mancabelli *et al*., highlighting *Firmicutes* (22–81%), *Bacteroides* (25.7%) and *Proteobacteria* (10.7%)^49,50^. We found that BS enriched classes such as *Clostridia* (32%), *Bacteroidia* (28%), while *Bacilli* (56%) were mostly enriched in RS which is in accordance with results reported by Danzeisen^51^ and Mohd^52^ *et al*. *Clostridiales* (32%) was the most abundant order in BS samples, while RS prefers *Lactobacillales* (57%) (Fig. 8). On the family level, RS and BS unraveled similar relative abundances for *Ruminococcacea* (BS: 19%, RS: 11%) while the family *Lactobacillaceae* (52%) was appreciably overrepresented with RS in comparison to BS specimens (19%) (Supplementary Table S3). These observations were not in accordance with the results of Lu *et al*.^53^, revealing *Clostridiaceae* as the most dominant family in broiler fecal samples. Further considerable differences were found in the distribution of the genera in comparison to other studies. As an example, Mohd *et al*. found that^52^, the most abundant genus was *Clostridium* (47–70%) and *Bacteroides* (2–20%) with low relative occurrence of *Lactobacillus* (<4%). We obtained relatively high read counts referring to the genus *Lactobacillus* (35.46%) samples achieving also low read counts for the genus *Clostridium* (<2%). *Bacteroides* also was more abundant in RF (5.64%) and BS sample (14.1%). Beside the methodological aspects the revealed taxa abundance differences can originate from a number of environmental (location and diet, etc.) and host (genetics and age, etc.) factors.

The discovery of the complex microbial communities depends heavily on the choice of the batch-effects of metagenome purification protocols often with a trade-off between successful taxonomic resolution. We unraveled tendentious effects of protocol batch-effects associated to approaches frequently used in the microbiome field. **a)** While examining the yield and the quality of the DNA purified from stool, we found that A_260_/A_280_ ratio proved to show a positive while the yield a negative correlation with successful downstream PCR amplification. We delineated that **b)** appreciable technical differences were generated by sample homogenization and DNA separation methods eventuating in solid β-diversity profiles while the lysis parameters represented only mild effects on compositional shifts. A strong positive **c)** association was also conveyed between community biodiversity and the application of robotic MagNa Pure24 or the conventional phenol-chlorophorm nucleic-acid based purification techniques. Across all other methods tested hereby the **d)** silica column-based applications seemed to be highly reproducible yielding the less diverse estimations. **e)** We demonstrated, that the species-level identification depends principally on the applied taxonomic database. **f)** We demonstrated that the cumulative bias exerted by the interaction of metagenomic protocol choices yields highly divergent results. **g)** We found that MagNa Pure 24 automated platform can be highly recommended for metagenomics solutions to achieve sufficient reproducibility while tracking large-scale microbiome shifts especially in multi-center consortia studies. Nevertheless, it proved to be prominent for the in-depth analysis of the microbiota communities as well.

All of our observations were taken by testing broiler stool samples on the basis of scarcity of standards to process livestock stool samples during metagenomic studies. We believe however, that some of our results can also serve as a guideline in optimizing DNA purification protocols for a wide range of samples.

## Materials and Methods

### Ethics approval

The study was approved and carried out in accordance with the local ethics committee’s guidelines of the University of Debrecen under the registration number: (DEMAB/12-7/2015).

### Sample collection

Broiler (*Gallus gallus domesticus*, Ross 308 hybrid) fecal samples were collected from the premise of Nagisz Zrt., Hungary, Hajdú-Bihar County; 4181, Nádudvar; Fő út 119. Individual fecal samples were collected (5 g of each) from eight randomly selected poultry; 4 pullets (female) and 4 cockerels (male). The freshly collected stool samples were stored in specific, sterile, DNase free stool transportation bowls and stored at 4 °C for maximum 3 hours. A composite fecal sample was made by pooling the individually collected feces and thoroughly mixed.

### Sample homogenization

Prior to lysis raw stool specimens (RS) were either homogenized via stirring manually with sterile stirrers or bacterial suspensions (BS) were obtained through multiple feces washing steps (Fig. 1). In the case of RS samples 250 mg raw specimens were further processed. To obtain BS 15-15 g specimens from different portions of the broiler stool specimens were transferred into 50 ml sterile, safe-lock-cap falcon tubes and 15-15 ml of PBS buffers were added. Samples were homogenized for 15 min (vortex at 350 RPM) and centrifuged for 5 min at 500 × g. These washing steps were repeated three times. Supernatants were collected and centrifuged at 13.000 × g for 20 minutes to pellet the bacterial cells. Finally, the supernatants were discarded and the pellets were dissolved in 3 ml PBS. And in every case 250 µl of BS was used for metagenomic DNA purification.

### Cell lysis

In the case of bead-beating three different, commercially available bead mills (Fig. 1) were used such as (0.5 mm Glass) PowerBead Tubes; Qiagen, Hilden, Germany (L1.1, L1.2), LightCycler® SeptiFast Lysis kit; Roche Diagnostics, Risch-Rotkreuz Switzerland (L1.3, L1.4), and MagNa Lyser Green Beads; Roche Diagnostics (L1.5, L1.6). For grinding two sample agitators; (L1.1, L1.3, L1.5) a standard laboratory vortex (10 min at 1800 x rpm) and the MagNa Lyser Instrument (L1.2, L1.4, L1.6), Roche Applied Sciences; Penzberg, Germany (30 sec at 5000 x rpm) were used. In the case of bead-beating 800 µl PBS was added to the specimens. Performing chemical lysis a lysis mixture of 500 µl lysis buffer and 60 µl proteinase K was prepared. To perform chemical lysis, commercially available ATL - Tissue Lysis Buffer (Qiagen Hilden, Germany) and BLB - Bacterial Lysis Buffer (Roche Applied Sciences) lysis buffers were used according to manufacturer’s instructions (L2.1, L2.2, L2.5) or for overnight incubation at 56 °C (L2.3, L2.4).

### DNA purification techniques

For magnetic bead-based purifications the automated MagNa Pure 24 System (Roche Applied Science) was used (P4.1, P4.2) by testing two protocols; Pathogen 200 (P4.1) with 200 µL initial sample volumes and Pathogen 1000 (P4.2) with 500 µL initial sample volumes with a universal reagent kit. Manual DNA separations were carried out via commercial (P5) and conventional (P6) methods. Applying manual commercial DNA isolation approaches; the QIAamp DNA Stool Mini kit, (P5.1), QIAmp Power Fecal kit (P5.2), and Qiagen DNeasy Power Soil kit (P5.3) (Qiagen Hilden, Germany) were used according to the manufacturer’s protocol. In the case of the conventional DNA purification technique (P6) bacterial DNA was isolated using phenol/chloroform/isoamyl alcohol method. Briefly, 800 µl of sample lysates was added to 800 µL of Ultrapure^TM^ phenol:chloroform:isoamyl alcohol (25:24:1) mixture (Thermo Fisher Scientific, Maryland, USA), and vortexed thoroughly for approximately 20 seconds. After incubation at room temperature for 3 minutes the lysate was centrifuged for 10 minutes at 16.000 × g. The upper aqueous phase was carefully removed and transferred into a new Eppendorf tube. For ethanol (EtOH) precipitation, 1 µl of 20 µg/µl glycogen, 7.5 M NH_4_OAc (ammonium acetate in 0.5 × volume of the sample) and 2.5X volume of 100% EtOH were added to the supernatant. The sample was placed at −20 °C overnight to precipitate the DNA from the sample. The sample was then centrifuged for 30 minutes at 16.000 g at 4 °C to pellet the DNA. The supernatant was carefully removed without disturbing the pellet. 500 µl 70% EtOH was added to the sample and shaken by hand approximately for 20 seconds. The sample was centrifuged at 4 °C for 5 minutes at 16.000 x g and the supernatant was carefully removed. This washing step was repeated 2 times. The DNA pellet was dried at room temperature under laminar air flow, and then the DNA pellet was dissolved in 30 µl of nuclease free water.

### Negative and positive controls

To minimize infections and laboratory contamination sterile surgical gloves and face masks (for collecting samples) were used and all DNA extraction steps were performed with sterile or sterilized equipments in a class II laminar air-flow cabinet. Negative isolation control (NIC) experiments were simultaneously conducted by substituting samples with PCR grade water. Elutes of the NIC samples were conveyed for V3-V4 amplicon - PCR and indexing performed under DNA free UV sterilized AirClean® PCR workstations/cabinets. At each stage the PCR clean-ups of the library preparation NIC amplicons were also validated on 4200 Tape Station System (G2991AA, Agilent Technologies; Santa Clara, California, United States) by Agilent D1000 ScreenTapes (5067–5365) and Agilent Genomic DNA reagents. Host background nucleic acid contaminations were also detected by conducting real-time PCR reactions using GAPDH assay on eluted gDNAs. For measuring the overall quality of Illumina MiSeq paired-end (PE, 2 × 301 nt) sequencing runs 5% PhiX spike-in quality control (PhiX Control Kit v3 - FC-110-3001) was used.

### Quality check

DNA concentrations were determined fluorometrically using Qubit® Fluorometric Quantitation dsDNA assay kit (Thermo Fisher Scientific, Waltham, Massachusetts, United States) on a Clariostar microplate reader (BMG Labtech, Ortenberg, Germany). All samples were diluted to 5 ng/µl with PCR-grade water. Purity was assessed by measuring the absorbance at 260 and 280 nm wavelengths using Nanodrop 2000 Spectrophotometer (Thermo Fisher Scientific, Maryland, USA). The optimal values of the absorbance ratios were expected to be in the range of 1.7–2.0 (A_260_/A_280_) and 1.8–2.2 (A_260_/A_230_). DNA integrity (DIN) was estimated automatically by running 1 µl of the gDNA samples on a 4200 TapeStation System, (G2991AA, Agilent Technologies; Santa Clara, California, United States) by using Genomic DNA ScreenTapes (5067–5365) and Agilent Genomic DNA reagents. The purified DNA samples were stored at −20 °C. For data analysis Analysis of Variance (ANOVA) test with Bonferroni corrections were conducted to compare the yield and purity of the nucleic acids such as the representative DIN values associated with the different technical variables.

### Library construction

Standard library preparation was performed according to Illumina (San Diego, California, United States) 16S Metagenomic Sequencing Library Preparation protocol (15044223 Rev. B). The V3 and V4 hypervariable regions of bacterial 16S rRNA gene were sequenced with Illumina MiSeq benchtop sequencer generating amplicons of ~460 by using the universal primer set: 341F-5′ CCTACGGGNGGCWGCAG 3′ and 785R-5′ GACTACHVGGGTATCTAATCC 3′ primers flanked by Illumina overhang adapter sequences (forward overhang: 5′ TCGTCGGCAGCGTCAGATGTGTATAAGAGACAG 3′, reverse overhang: 5′ GTCTCGTGGGCTCGGAGATGTGTATAAGAGACAG 3′) (Sigma Aldrich, Missouri, US). After completion of the amplicon PCR with 2 x KAPA HiFi HotStart ReadyMix dual indexing of the 96 samples (see positions of indexed samples in Supplementary Fig. S1 with adaptor sequences (i7-N7xx-12 items, i5-S5xx-8 items)) was performed using the Illumina Nextera XT Index Kit (FC-131-1001/2). PCR cleanups and amplicon size selections were carried out with KAPA Pure Beads (KAPA Biosystems) based on the technical data sheet (KR1245 – v3.16) of the manufacturer resulting in final ~580–630 bp libraries. Every time, verifications were done with PCR Agilent D1000 screen tapes (5067–5582) and D1000 Reagents (5067–5583). The 16S amplicon libraries for each sample were quantified with qPCR, normalized with respect to amplicon sizes and pooled into a single library in equal molar quantities. Finally, 5 µl of pooled 4 nM DNA library pool was prepared for sequencing on Illumina MiSeq platform. The library pool was denatured with 0.2 M NaOH and diluted to 10 pM final concentration. Sequencing was carried out with MiSeq Reagent Kit v3–618 cycle (MS-102–3003) following manufacturer’s protocols (Illumina, Inc., San Diego, CA, USA).

### Bead-based re-purification of isolated gDNA samples after unsuccessful amplicon PCR

In the case of impaired amplicon PCR (missing ~550 bp TapeStation traces) bead-based amplicon purifications were done in the second place on isolated gDNA samples for inhibitor removal and retention of smaller DNA fragments. In the case of 16 samples (A3, D1, F4, F6, F11, F12, G1, G4, G6, G8, H1, H3, H8, H9, H10, H11) KAPA Pure Beads were applied in a sample to bead ratio of 1:3, and the purified DNA was eluted in 25 μL of Tris Buffer (10 mM, pH 8.0). After DNA purification, amplicon PCR was repeated.

### Sequencing read preparation for downstream analysis

Paired end reads were demultiplexed by the integrated software of the Illumina MiSeq sequencing machine^54^. The FastQ files were imported into the Qiime 2 (ver 2019.7) pipeline (https://qiime2.org/) according to the “Atacama Soil microbiome” tutorial^54^. The presence of adapter sequences (CTGTCTCTTATACACATCT) was checked and trimmed from the 3′ end of the reads with Cutadapt Software integrated in the Qiime 2 pipeline. Quality trimming was performed by using the DADA2 software^55^ and the denoising parameters were set as follows: for the forward reads 13 bases were cropped from the start and the length was set to 290 bases; for the reverse reads 8 bases were cropped from the start of the reads and the length was set to 280 bases.

### Alignment

Multiple sequence alignment was performed with the Mafft software^56^, and reads were taxonomically classified using Naïve Bayesian classifier trained with the Greengenes (ver13_8) and Silva (ver132)^57^ reference databases by selecting mapping points according to the forward-reverse primer set that was used for amplifying the 16S V3-V4 regions of the bacterial community (341 F, 806 R). Phylogenetic tree was constructed with FastTree plugin^58^.

### Biodiversity analysis

Alpha and beta diversity tests were performed in the QIIME2 pipeline. For sample normalization a 25 000 read depth was set. However, alpha rarefaction analysis showed that 5 000 reads could be enough to maximize the diversity metrics. For alpha diversity Shannon’s index^59^, Faith’s phylogenetic diversity index^60^, Simpson evenness^61^, and Chao1 index^62^ was calculated in the QIIME pipeline. Beta diversity analysis involved measuring weighted/unweighted UNIFRAC distances^63^ and Bray-Curtis dissimilarities^64^. For visualization of beta diversity matrices PCoA plots were generated using the Emperor^65^ plugin. Alpha diversity differences were measured using the Kruskal-Wallis test. Beta diversity group significances were calculated with Permutational multivariate analysis of variance (PERMANOVA) pseudo F statistical test^66^.

### Data visualization

For data mining (preparation) the QIIME2 pipeline was used. QIIME artifact files were exported from the pipeline resulting BIOM files that were converted to TSV files which were used with different visualization packages. Heatmaps were generated in Python (ver3.6.5) with Seaborn package^67^. Bar charts, boxplots, pie charts, polar plots, bubble plots were constructed with R programming language^68^ using the ggplot2 package^69^. Differential heat tree was created with the metacoder R package^70^. In the case of community heat-trees significant differences were determined using a Wilcox rank-sum test followed by a Benjamin-Hochberg (FDR) correction for multiple comparisons^70^.

## Supplementary information

Supplementary Information

## Data Availability

All sequence data used in the analyses were deposited in the Sequence read Archive (SRA) (http://www.ncbi.nlm.nih.gov/sra) under PRJNA533250. Sample IDs, meta data and corresponding accession numbers are summarized in Supplementary Fig. S1.
