# Supplementary material for: Tendentious effects of automated and manual metagenomic DNA purification protocols on broiler gut microbiome taxonomic profiling
Source: Sci Rep. 2020 Feb 25;10:3419. doi: 10.1038/s41598-020-60304-y (PMC7042355; doi:10.1038/s41598-020-60304-y)
Supplement: Supplementary file 1 — Supplementary Information [file 41598_2020_60304_MOESM1_ESM.pdf]

# Tendentious effects of automated and manual metagenomic DNA purification protocols on broiler gut microbiome taxonomic profiling

Gabor Fidler[1†], Emese Tolnai[1†], Aniko Stagel[1], Judit Remenyik[2], Laszlo Stundl[2], Ferenc Gal[2], Sandor Biro[1], Melinda Paholcsek[1*]

**Author's details:**

[1]Department of Human Genetics, Faculty of Medicine, University of Debrecen, Debrecen, Hungary. [2]Institute of Food Technology, Faculty of Agricultural and Food Sciences and Environmental Management, University of Debrecen, Debrecen, Hungary. [†] Both authors contributed equally to this work. *corresponding author

| | A | B | C | D | E | F | G | H |
|---|---|---|---|---|---|---|---|---|
| **1** | BS 1.1 4.1 | BS 1.5 4.2 | BS 1.6+2.1 4.1 | RS 2.2 4.2 | BS 2.2 4.2 | BS 1.2 6 | BS 2.2 1.3 6 | RS 2.2 6 |
| **2** | BS 1.2 4.1 | BS 2.2 4.2 | BS 2.2 1.2 4.1 | RS 2.2 4.2 | BS 2.5 5.1 | BS 1.3 6 | BS 1.1 2.2 6 | RS 2.3 6 |
| **3** | BS 1.3 4.1 | BS 2.5 4.2 | BS 2.2 1.4 4.1 | RS 2.3 4.2 | RS 2.5 5.1 | BS 1.4 6 | BS 1.3 2.2 6 | RS 2.4 6 |
| **4** | BS 1.4 4.1 | BS 2.2 4.2 | BS 2.2 4.1 | RS 2.1 1.4 4.1 | BS 2.1 1.1 5.2 | BS 2.5 6 | BS 1.1+2.1 6 | RS 2.1 1.1 6 |
| **5** | BS 1.5 4.1 | BS 2.1 1.2 4.1 | BS 2.4 4.1 | RS 1.2 2.1 4.1 | BS 2.1 1.2 5.2 | BS 2.2 6 | BS 1.3+2.1 6 | RS 2.1 1.3 6 |
| **6** | BS 1.6 4.1 | BS 2.1 1.4 4.1 | RS 2.1 1.2 4.2 | RS 1.4 2.1 4.1 | RS 2.1 1.1 5.2 | BS 2.3 6 | BS 1.1+2.2 6 | RS 1.1 2.1 6 |
| **7** | BS 2.1 4.1 | BS 2.1 1.6 4.1 | RS 2.1 1.4 4.2 | RS 1.2+2.1 4.1 | RS 2.1 1.2 5.2 | BS 2.4 6 | BS 1.3+2.2 6 | RS 1.1 2.1 6 |
| **8** | BS 2.2 4.1 | BS 1.2 2.1 4.1 | RS 1.2 2.1 4.2 | RS 1.4 2.1 4.1 | BS 2.1 1.1 5.3 | BS 2.1 1.1 6 | RS 1.1 6 | RS 2.2 1.1 6 |
| **9** | BS 1.1 4.2 | BS 1.4 2.1 4.1 | RS 1.4 2.1 4.2 | RS 2.2 4.1 | BS 2.1 1.2 5.3 | BS 2.1 1.3 6 | RS 1.2 6 | RS 2.2 1.3 6 |
| **10** | BS 1.2 4.2 | BS 1.6 2.1 4.1 | RS 1.2+2.1 4.1 | RS 2.2 4.1 | RS 2.1 1.1 5.3 | BS 1.1 2.1 6 | RS 1.3 6 | RS 1.1 2.2 6 |
| **11** | BS 1.3 4.2 | BS 1.2+2.1 4.1 | RS 1.4+2.1 4.1 | RS 2.4 4.1 | RS 2.1 1.2 5.3 | BS 1.3 2.1 6 | RS 1.4 6 | RS 1.3 2.2 6 |
| **12** | BS 1.4 4.2 | BS 1.4+2.1 4.1 | RS 2.2 1.2 4.2 | BS 2.1 4.2 | BS 1.1 6 | BS 2.2 1.1 6 | RS 2.1 6 | RS 1.1+2.1 6 |

**Supplementary Figure S1.** Experimental design indicating protocol manipulations of the 96 methods.

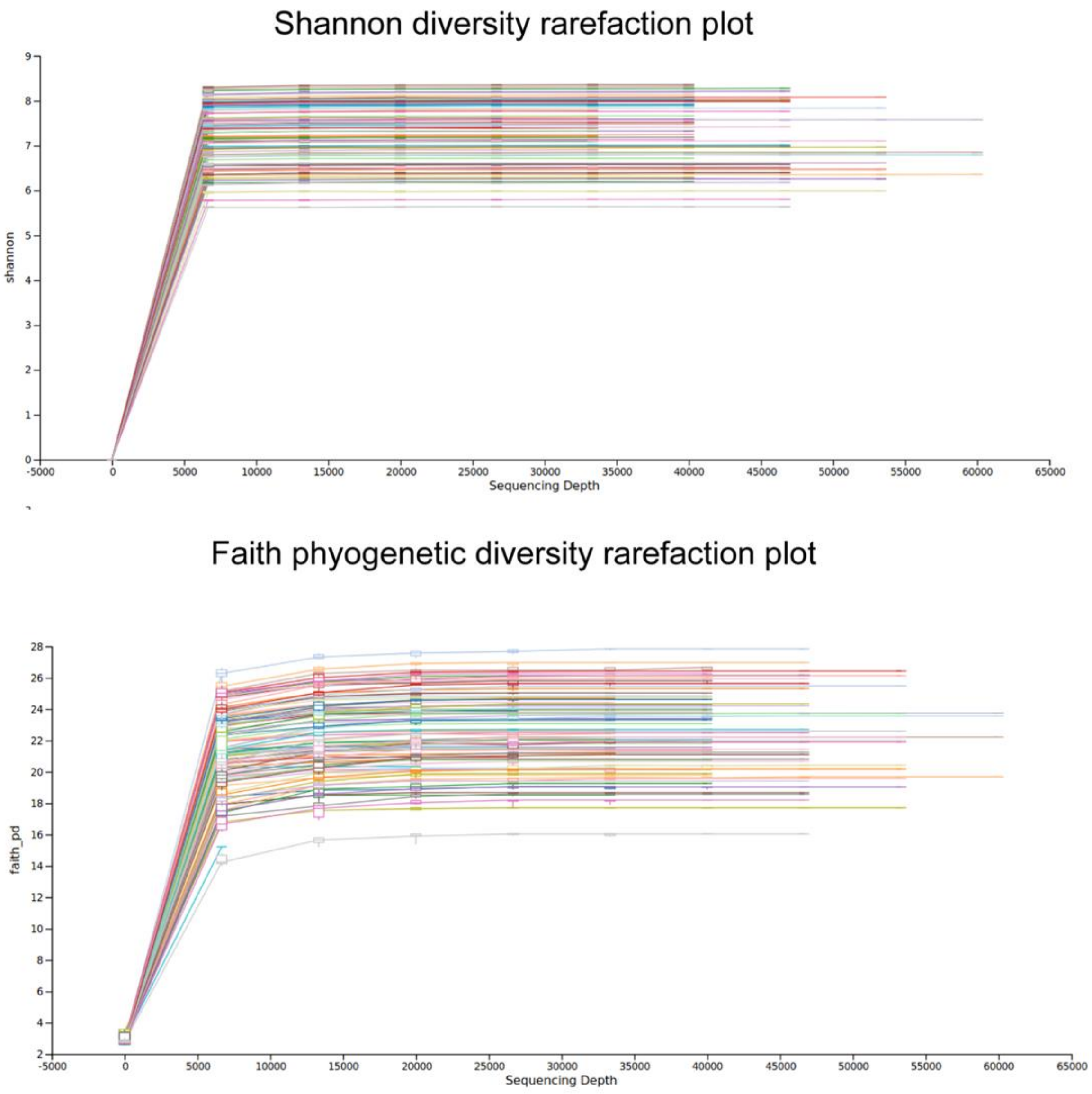


**Supplementary Figure S2.** Sequencing read depth in correlation with alpha-diversity. Plots show, that 5000 reads per sample are enough to maximize the Shannon’s and Faith phylogenetic diversity. Increasing the read number will not affect the overall diversity significantly, but can cause the loss of samples.

| Stirring (RS) | | | | | | | |
|---|---|---|---|---|---|---|---|
| **S1(P4.1)** | **S2 (P4.2)** | **S3 (P5.1)** | **S4 (P5.2)** | **S5 (P5.3)** | **S6 (P6-L1)** | **S7 (P6-L2)** | **S8 (P6-L3)** |
| Roche MagNa Pure 24 Pathogen 200 | Roche MagNa Pure 24 Pathogen 1000 | QIAmp DNA Stool Mini kit | QIAmp Power Fecal kit | DNeasy Power Soil kit | Conventional | | |
| D11, D10, D9 | D2, D1, C12 | E3 | E6, E7 | E10, E11 | G8, G9, G10, G11 | G12, H1, H2, H3 | H4, H5, H6, H7, H8, H9, H10, H11, H12 |
| **Washing (BS)** | | | | | | | |
| **S9(P4.1)** | **S10 (P4.2)** | **S11 (P5.1)** | **S12 (P5.2)** | **S13 (P5.3)** | **S14 (P6-L1)** | **S15 (P6-L2)** | **S16 (P6-L3)** |
| Roche MagNa Pure 24 Pathogen 200 | Roche MagNa Pure 24 Pathogen 1000 | QIAmp DNA Stool Mini kit | QIAmp Power Fecal kit | DNeasy Power Soil kit | Conventional DNA precipitation | | |
| A8, C5, C4 | B4, E1, B2 | E2 | E4, E5 | E8, E9 | E12, F1, F2, F3 | F4, F5, F6, F7 | F8, F9, F10, F11, F12, G1, G2, G3, G4, G5, G6, G7 |

**Supplementary Table S1.** Representation of plate positions associated with standard metagenome DNA isolation methods.

| | Ranking of methods according to: | | |
| --- | --- | --- | --- |
| Sample Id | (C) Shannon's Diversity Index | (B) Obsereved OTUs | (A) DNA Concentration ng/µL |
| A1 | 64 | 76 | 67 |
| A2 | 65 | 82 | 51 |
| A3 | 38 | 39 | 75 |
| A4 | 45 | 42 | 71 |
| A5 | 57 | 59 | 86 |
| A6 | 56 | 41 | 84 |
| A7 | 44 | 1 | 50 |
| A8 | 84 | 68 | 70 |
| A9 | 47 | 55 | 38 |
| A10 | 50 | 64 | 26 |
| A11 | 35 | 45 | 24 |
| A12 | 70 | 70 | 48 |
| B1 | 37 | 13 | 33 |
| B2 | 69 | 74 | 82 |
| B3 | 31 | 31 | 9 |
| B4 | 34 | 15 | 13 |
| B5 | 26 | 40 | 37 |

| | | | |
|---|---|---|---|
| B6 | 30 | 46 | 35 |
| B7 | 16 | 11 | 36 |
| B8 | 21 | 28 | 15 |
| B9 | 5 | 7 | 29 |
| B10 | 17 | 19 | 19 |
| B11 | 13 | 12 | 40 |
| B12 | 28 | 36 | 44 |
| C1 | 3 | 2 | 61 |
| C2 | 4 | 5 | 58 |
| C3 | 6 | 6 | 60 |
| C4 | 2 | 3 | 59 |
| C5 | 1 | 4 | 76 |
| C6 | 66 | 38 | 62 |
| C7 | 53 | 9 | 53 |
| C8 | 58 | 47 | 52 |
| C9 | 54 | 35 | 46 |
| C10 | 61 | 51 | 56 |
| C11 | 51 | 50 | 45 |
| C12 | 41 | 54 | 80 |
| D1 | 59 | 58 | 72 |
| D2 | 39 | 72 | 74 |
| D3 | 49 | 80 | 78 |

| | | | |
|---|---|---|---|
| D4 | 60 | 67 | 68 |
| D5 | 40 | 52 | 63 |
| D6 | 62 | 69 | 49 |
| D7 | 55 | 65 | 73 |
| D8 | 52 | 83 | 66 |
| D9 | 42 | 62 | 77 |
| D10 | 7 | 48 | 81 |
| D11 | 46 | 49 | 79 |
| D12 | 48 | 73 | 30 |
| E1 | 43 | 37 | 64 |
| E2 | 80 | 84 | 85 |
| E3 | 83 | 81 | 89 |
| E4 | 81 | 85 | 93 |
| E5 | 79 | 63 | 90 |
| E6 | 90 | 91 | 92 |
| E7 | 94 | 93 | 88 |
| E8 | 68 | 60 | 87 |
| E9 | 76 | 57 | 95 |
| E10 | 73 | 78 | 91 |
| E11 | 82 | 79 | 94 |
| E12 | 32 | 34 | 96 |
| F1 | 33 | 44 | 39 |

| | | | |
|---|---|---|---|
| F2 | 24 | 29 | 41 |
| F3 | 74 | 56 | 18 |
| F4 | 9 | 8 | 83 |
| F5 | 10 | 10 | 42 |
| F6 | 12 | 23 | 20 |
| F7 | 14 | 18 | 16 |
| F8 | 18 | 22 | 31 |
| F9 | 20 | 32 | 22 |
| F10 | 25 | 16 | 12 |
| F11 | 19 | 20 | 11 |
| F12 | 29 | 14 | 21 |
| G1 | 23 | 25 | 27 |
| G2 | 8 | 21 | 43 |
| G3 | 15 | 24 | 32 |
| G4 | 67 | 26 | 69 |
| G5 | 11 | 27 | 17 |
| G6 | 22 | 33 | 10 |
| G7 | 36 | 87 | 34 |
| G8 | 85 | 30 | 25 |
| G9 | 89 | 88 | 28 |
| G10 | 72 | 86 | 57 |
| G11 | 88 | 89 | 4 |

| | | | |
|---|---|---|---|
| G12 | 96 | 96 | 54 |
| H1 | 92 | 94 | 23 |
| H2 | 91 | 90 | 2 |
| H3 | 93 | 92 | 3 |
| H4 | 86 | 75 | 5 |
| H5 | 75 | 43 | 65 |
| H6 | 95 | 95 | 47 |
| H7 | 63 | 53 | 8 |
| H8 | 71 | 61 | 6 |
| H9 | 77 | 66 | 14 |
| H10 | 78 | 77 | 7 |
| H11 | 27 | 17 | 1 |
| H12 | 87 | 71 | 55 |

**Supplementary Table S2.** Rankings of the 96 different metagenomic DNA extractions to observed ASV numbers (column B) in relation of DNA yield (column A) and Shannon's diversity indexes (column C).

**Bacterial Suspension (BS)**

| **Phylum** | | **Class** | | **Order** | | **Family** | | **Genus** | | **Species** | |
|---|---|---|---|---|---|---|---|---|---|---|---|
| *Actinobacteria* | 1.1020979882 | *Clostridia* | 32.100664176 | *Clostridiales* | 32.08896673 | *Ruminococcaceae* | 19.5283391 | *Lactobacillus* | 18.9450832 | *Unknown* | 86.88301289 |
| *Bacteroidetes* | 28.688718888 | *Bacteroidia* | 28.686742912 | *Bacteroidales* | 28.62921766 | *Lactobacillaceae* | 18.9450832 | *Bacteroides* | 14.1091148 | *Lactobacillus salivarius* | 2.677829001 |
| *Cyanobacteria* | 0.6418643311 | *Bacilli* | 25.548865867 | *Lactobacillales* | 25.18859237 | *Bacteroidaceae* | 14.1091148 | *Unknown* | 9.52379486 | *Bacteroides gallinaceum* | 2.617802658 |
| *Epsilonbacteraeota* | 1.9119322117 | *Gammaproteobacteria* | 5.7824991659 | *Enterobacteriales* | 4.643268449 | *Lachnospiraceae* | 7.42733638 | *Alistipes* | 6.25301334 | *Lactobacillus aviarius* | 2.202312630 |
| *Firmicutes* | 59.941162436 | *Campylobacteria* | 1.9118005247 | *Campylobacterales* | 1.911800524 | *Rikenellaceae* | 6.25316725 | *Streptococcus* | 4.98307141 | *Bacteroides uniformis* | 1.50146660 |
| *Lentisphaerae* | 0.0048870965 | *Erysipelotrichia* | 1.7904010952 | *Erysipelotrichales* | 1.790401095 | *Streptococcaceae* | 4.99869365 | *Escherichia-Shigella* | 4.62510663 | *Bacteroides sp. SB5* | 0.80546886 |
| *Patescibacteria* | 0.00019240537 | *Verrucomicrobiae* | 0.9621145279 | *Verrucomicrobiales* | 0.962114527 | *Enterobacteriaceae* | 4.64326844 | *Ruminococcaceae UCG-014* | 4.16882947 | *Anaeromassilibacillus senegalensis* | 0.66279086 |
| *Proteobacteria* | 6.57260609232 | *Actinobacteria* | 0.7717232831 | *Gastranaerophilales* | 0.631623339 | *Barnesiellaceae* | 3.30529667 | *Faecalibacterium* | 3.78900894 | *Campylobacter jejuni* | 0.638434020 |
| *Tenericutes* | 0.17435775085 | *Melainabacteria* | 0.6316233393 | *Pseudomonadales* | 0.629122241 | *Unknown* | 3.09835970 | *Barnesiella* | 2.41613723 | *Lactobacillus vaginalis* | 0.214978958 |
| *Verrucomicrobia* | 0.96218079948 | *Negativicutes* | 0.4939475362 | *Corynebacteriales* | 0.575829623 | *Prevotellaceae* | 2.18026449 | *Prevotellaceae UCG-001* | 2.17964883 | *Mordavella massiliensis* | 0.16003 |

**Raw Stool Specimens (RS)**

| **Phylum** | | **Class** | | **Order** | | **Family** | | **Genus** | | **Species** | |
|---|---|---|---|---|---|---|---|---|---|---|---|
| *Actinobacteria* | 1.3776821185 | *Bacilli* | 56.004035651 | *Lactobacillales* | 55.86466177 | *Lactobacillaceae* | 52.0731793 | *Lactobacillus* | 52.0731793 | *Unknown* | 89.3344130 |
| *Bacteroidetes* | 12.627088818 | *Clostridia* | 23.436784551 | *Clostridiales* | 23.43405173 | *Ruminococcaceae* | 11.4854888 | *Unknown* | 5.80891780 | *Lactobacillus salivarius* | 3.21000502 |
| *Cyanobacteria* | 0.5814475562 | *Bacteroidia* | 12.626525430 | *Bacteroidales* | 12.42518902 | *Lachnospiraceae* | 6.41248084 | *Bacteroides* | 5.64176955 | *Lactobacillus aviarius* | 2.20901172 |
| *Epsilonbacteraeota* | 0.5006863033 | *Gammaproteobacteria* | 2.4160368629 | *Erysipelotrichales* | 1.603775531 | *Bacteroidaceae* | 5.64176955 | *Barnesiella* | 3.61200857 | *Lactobacillus vaginalis* | 1.68531398 |
| *Firmicutes* | 81.435174314 | *Erysipelotrichia* | 1.6037755316 | *Enterobacteriales* | 1.349623177 | *Barnesiellaceae* | 3.84374064 | *Streptococcus* | 3.51223272 | *Phaseolus acutifolius* | 0.43412813 |
| *Fusobacteria* | 0.0011154869 | *Actinobacteria* | 1.2297694558 | *Pseudomonadales* | 0.722580184 | *Streptococcaceae* | 3.51245580 | *Romboutsia* | 3.21876120 | *Bacteroides sp. SB5* | 0.39084917 |
| *Lentisphaerae* | 0.0007250664 | *Campylobacteria* | 0.5006639639 | *Micrococcales* | 0.643161059 | *Peptostreptococcaceae* | 3.38306509 | *Faecalibacterium* | 2.89338819 | *Lactobacillus oris* | 0.26764914 |
| *Patescibacteria* | 0.0002788717 | *Oxyphotobacteria* | 0.4671450776 | *Campylobacterales* | 0.500663963 | *Unknown* | 2.11146674 | *Ruminococcaceae UCG-014* | 1.85848560 | *Bacteroides gallinaceum* | 0.21159842 |
| *Proteobacteria* | 3.06876028 | *Negativicutes* | 0.3863316562 | *Chloroplast* | 0.467145077 | *Erysipelotrichaceae* | 1.60377553 | *[Ruminococcus] torques* | 1.64577174 | *Lactobacillus pontis* | 0.19152055 |
| *Tenericutes* | 0.217966143 | *Deltaproteobacteria* | 0.3846585005 | *Corynebacteriales* | 0.463017960 | *Christensenellaceae* | 1.35726392 | *Subdoligranulum* | 1.42117847 | *Lactobacillus panis* | 0.17863725 |

**Supplementary Table S3.** Silva databases taxonomic data. Relative frequencies are shown for the ten most abundant taxa in Phylum, Class, Order, Family, Genus and Species level.

**Silva databases**

| | *Family (L5)* | | | *Genus (L6)* | | | *Species (L7)* | |
|---|---|---|---|---|---|---|---|---|
| Group | Identified | Not identified | Group | Identified | Not identified | Group | Identified | Not identified |
| BS | 99.49 | 0.51 | BS | 96.69 | 3.31 | BS | 14.42 | 85.58 |
| RF | 98.13 | 1.87 | RF | 97.41 | 2.59 | RF | 9.16 | 90.84 |
| L1 | 99.71 | 0.29 | L1 | 95.11 | 4.89 | L1 | 10.71 | 89.29 |
| L2 | 97.85 | 2.15 | L2 | 96.41 | 3.59 | L2 | 11.48 | 88.52 |
| L3 | 99.50 | 0.50 | L3 | 98.27 | 1.73 | L3 | 16.72 | 83.28 |
| P4 | 99.25 | 0.75 | P4 | 94.98 | 5.02 | P4 | 15.09 | 84.91 |
| P5 | 95.04 | 4.96 | P5 | 94.79 | 5.21 | P5 | 7.16 | 92.84 |
| P6 | 94.65 | 5.35 | P6 | 94.23 | 5.77 | P6 | 18.28 | 81.72 |
| **mean** | 97.95 | 2.05 | **mean** | 95.99 | 4.01 | **mean** | 12.88 | 87.12 |
| **SD** | 2.03 | 2.03 | **SD** | 1.42 | 1.42 | **SD** | 3.86 | 3.86 |

**Greengenes databases**

| | *Family (L5)* | | | *Genus (L6)* | | | *Species (L7)* | |
|---|---|---|---|---|---|---|---|---|
| Group | Identified | Not identified | Group | Identified | Not identified | Group | Identified | Not identified |
| BS | 88.81 | 11.19 | BS | 74.81 | 25.19 | BS | 44.49 | 55.51 |
| RF | 92.16 | 7.84 | RF | 81.99 | 18.01 | RF | 56.15 | 43.85 |
| L1 | 85.00 | 15.00 | L1 | 74.35 | 25.65 | L1 | 50.63 | 49.37 |
| L2 | 91.50 | 8.50 | L2 | 78.77 | 21.23 | L2 | 51.33 | 48.67 |
| L3 | 91.63 | 8.37 | L3 | 78.65 | 21.35 | L3 | 48.08 | 51.92 |
| P4 | 87.64 | 12.36 | P4 | 70.31 | 29.69 | P4 | 41.81 | 58.19 |
| P5 | 93.04 | 6.96 | P5 | 88.59 | 11.41 | P5 | 68.68 | 31.32 |
| P6 | 92.48 | 7.52 | P6 | 83.46 | 16.54 | P6 | 52.71 | 47.29 |
| **mean** | 90.28 | 9.72 | **mean** | 78.86 | 21.14 | **mean** | 51.74 | 48.26 |
| **SD** | 2.84 | 2.84 | **SD** | 5.80 | 5.80 | **SD** | 8.22 | 8.22 |

**Supplementary Table S4**. Table shows the percentages of the identified reads at certain taxonomic levels bound to protocol manipulations.
